# Generation of *Kcnma1*^*fl*^*-tdTomato*, a conditional deletion of the BK channel *α* subunit in mouse

**DOI:** 10.14814/phy2.12612

**Published:** 2015-11-04

**Authors:** Betsir G Zemen, Michael H Lai, Joshua P Whitt, Zulqarnain Khan, Guiling Zhao, Andrea L Meredith

**Affiliations:** 1 Department of Physiology, University of Maryland School of MedicineBaltimore, Maryland; 2 Center of BioMedical Engineering and Technology and Department of Physiology, University of Maryland School of MedicineBaltimore, Maryland

**Keywords:** BK channel, calcium-activated potassium channel, Cre-lox, *Kcnma1*, maxi K, *mSlo1*, potassium channel, red fluorescent protein, tandem dimer tomato

## Abstract

BK large conductance calcium-activated K^+^ channels (K_C__a_1.1) are expressed widely across many tissues, contributing to systemic regulation of cardiovascular, neurological, and other specialized physiological functions. The pore-forming *α* subunit is encoded by the *Kcnma1* gene, originally named *mSlo1* in mouse and *slowpoke* in *Drosophila*. Global deletion in mouse (*Kcnma1*^*−/−*^) produces a plethora of defects in neuron and muscle excitability, as well as other phenotypes related to channel function in nonexcitable cells. While homozygous null mice are viable, the ubiquitous loss of BK function has complicated the interpretation of phenotypes involving the interaction of multiple cell types which independently express BK channels. Here, we report the generation of a targeted allele for conditional inactivation of *Kcnma1* using the Cre-loxP system (*Kcnma1*^*fl*^*-tdTomato*). Cre-mediated recombination generates a null allele, and BK currents were not detectable in neurons and muscle cells from Nestin-Cre; *Kcnma1*^*fl/fl*^ and SM22*α*-Cre; *Kcnma1*^*fl/fl*^ mice, respectively. tdTomato expression was detected in Cre-expressing tissues, but not in Cre-negative controls. These data demonstrate the utility of *Kcnma1*^*fl*^*-tdTomato* for conditional deletion of the BK channel, facilitating the understanding of tissue-specific contributions to physiological function in vivo.

## Introduction

BK currents are produced by tetrameric assembly of four pore-forming *α* subunits, encoded by the *Kcnma1* gene (Butler et al. 1993). BK current properties are modulated by several nonobligatory *β* (*β*1–4) and *γ* accessory subunits (*γ*1–4) (Brenner et al. 2000a; Yan and Aldrich 2012), tuning current properties for diverse roles across a variety of tissues. In rodents, BK channels are found in brain (Tseng-Crank et al. 1994; Kang et al. 1996; Smith et al. 2002; Faber and Sah 2003; Sausbier et al. 2004; Brenner et al. 2005; Girouard et al. 2010), peripheral neurons (Scholz et al. 1998; Ramanathan et al. 1999), muscle (Tseng-Crank et al. 1994; McCobb et al. 1995; Nelson et al. 1995; Heppner et al. 1997), and nonexcitable cells such as glia, kidney, bone, and endothelium (Morita et al. 1997; Papassotiriou et al. 2000; Ransom and Sontheimer 2001; Filosa et al. 2006; Henney et al. 2009; Li et al. 2009). Despite the ubiquity of BK currents, mice carrying targeted mutations in the BK channel pore-forming *α* subunit, which do not produce functional BK currents, are viable (Meredith et al. 2004; Sausbier et al. 2004). In addition to these loss-of-function lines, a gain-of-function allele has been generated expressing a BK channel cDNA harboring the R207Q mutation, which enhances voltage-dependent gating (Montgomery and Meredith 2012). Additionally, loss-of-function deletions of the *β*1, *β*2, and *β*4 subunits have been generated (Brenner et al. 2000b, 2005; Martinez-Espinosa et al. 2014).

Global deletions of *Kcnma1* (*Kcnma1*^*−/−*^) have been indispensable for understanding how BK currents regulate cellular and integrated physiology in mammals, revealing the essential function of BK channels in neurons, muscle, and nonexcitable cells in vivo. *Kcnma1*^*−/−*^ mice have alterations in circadian rhythm, heart rate, blood pressure, urination, locomotion, reproductive function, neurovascular coupling, hearing, airway constriction, insulin secretion, and neurological learning behaviors (Meredith et al. 2004, 2006; Ruttiger et al. 2004; Sausbier et al. 2004, 2005, 2007; Werner et al. 2005; Filosa et al. 2006; Pyott et al. 2007; Dufer et al. 2011; Typlt et al. 2013; Lai et al. 2014). These mice were further used to demonstrate that BK channels are the targets of a fungal neurotoxin that causes Ryegrass Staggers (Imlach et al. 2008) and can be localized to intracellular organelles and to complexes containing Ca^2+^ channels (Indriati et al. 2013; Singh et al. 2013; Li et al. 2014; Cao et al. 2015). However, the ubiquitous loss of BK function in several systems has made interpretation of phenotypes challenging. For example, the ataxia in *Kcnma1*^*−/−*^ mice initially confounded analysis of circadian rhythms in locomotor activity (Meredith et al. 2006) and the contribution of BK channels to vascular hypertension was complicated by hyperaldosteronism (Sausbier et al. 2005). Furthermore, aspects of cardiac, bladder, and renal function are compensated in *Kcnma1*^*−/−*^ mice (Rieg et al. 2007; Sprossmann et al. 2009; Lai et al. 2014). Thus, to provide a higher resolution picture for the distinct contributions of BK channels in particular tissues to changes in physiology, we generated a targeted floxed allele, *Kcnma1*^*fl*^*-tdTomato*. BK currents and *tdTomato* fluorescence were evaluated in neurons and smooth muscle, two tissue types exhibiting the highest endogenous expression levels, using Nestin-Cre and SM22*α*-Cre drivers, respectively (Tronche et al. 1999; Lepore et al. 2005).

## Materials and Methods

### Generation of Kcnma1^fl^-tdTomato mice

A targeting vector was created that contained 10.95 kb of *Kcnma1* genomic sequence flanking exon 2 (B6 BAC clone RP23: 64P21) subcloned into pSP72, containing an ampicillin selection cassette (Ingenious Targeting Laboratory, Ronkonkoma, NY). A Lox71 site was subcloned 3′ of the 6.02-kb long homology arm (LA), 312-bp upstream of exon 2. A mini-gene was generated consisting of a Lox66 site, 255-bp intron sequence, 33 bp of exon 2, the 2A-tdTomato sequence, followed by a bovine growth hormone polyadenylation sequence (BGHpA). The final 2A-tdTomato cassette was inserted into the targeting vector using MluI and SalI sites. The short homology arm (SA) extends 3.77 kb 3′ to the mini gene. A pGK-gb2 FRT-flanked neomycin resistance cassette was subcloned 327-bp downstream of exon 2, 5′ to a 2A-tdTomato cassette in reverse orientation. The targeting vector was confirmed by restriction analysis and sequencing after each modification. The total targeting vector size was 17.13 kb, and the construct was linearized with NotI for electroporation into BA1 (C57BL/6 × 129/SvEv) embryonic stem cells (Ingenious Targeting Laboratory).

**Figure 1:**
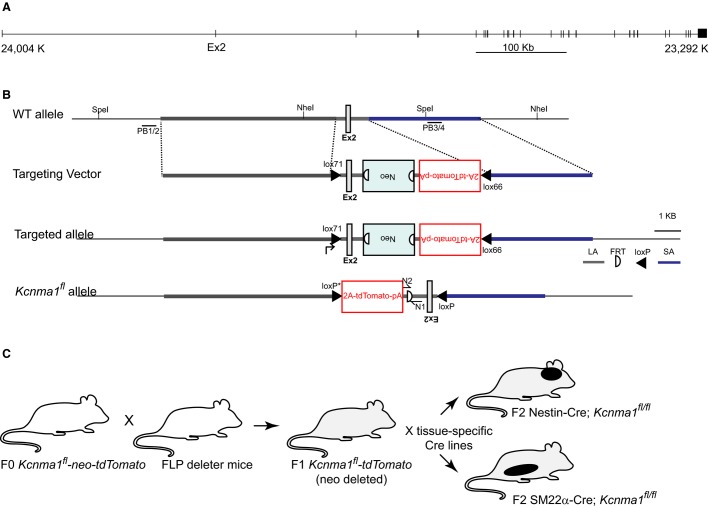
*Kcnma1* gene structure and conditional inactivation strategy. (A) *Mus musculus Kcnma1*, encoded on the complement strand of chromosome 14 (24,004,951–23,292,291). The 29 exons comprising the coding sequence are arranged to scale in the genomic context, presented 5′ to 3′ for ease of viewing. (B) Wild-type (WT) allele in the region of exon 2. The targeting vector was electroporated into ES cells, and the targeted allele for correctly integrated clones is depicted after homologous recombination. After deletion of the neocassette, Cre expression inactivates *Kcnma1* by inverting exon 2 and bringing the 2A-tdTomato cassette into frame. (C) Breeding scheme for generating neuronal and smooth muscle conditional tissue-specific deletions of the BK channel.

Correctly targeted clones were identified by PCR and confirmed by Southern blot analysis, using an external probe (PB1/2) on SpeI-digested DNA (WT: 10.3 kb; *Kcnma1*^*fl*^*-tdTomato*: 8.2 kb) and an internal probe on NheI-digested DNA (WT: 7.8 kb; *Kcnma1*^*fl*^*-tdTomato*: 11.5 kb; Fig.1B). Three clones were microinjected into C57BL/6 blastocysts, and the resulting chimeric animals from clone #712 with a high percentage of agouti coat color were mated to a FLP deleter strain: B6.SJL-Tg(ACTFLPe)9205Dym/J (Jackson Labs, Bar Harbor, MA) (Rodriguez et al. 2000). Mice were screened for neodeletion by PCR with neodirected primers N1: 5′-CAAAGGGGGTTTGCTTGTGAGAGG-3′ and N2: 5′-CATGAGCGTGTGCCTAAACGCA-3′ (Fig.1B), which amplified a 642-bp product from the FLP-recombined, neodeleted allele and a 577-bp product from WT controls. Neodeleted progeny were mated to SM22*α*-Cre (Lepore et al. 2005) or Nestin-Cre [B6.Cg(SJL)-Tg(Nes-cre)1Kln/J, Jackson Labs] (Tronche et al. 1999). Mice were routinely genotyped with “Kcnma1-3 WT,” “tdRFP,” and “Cre” probes (Transnetyx, Cordova, TN). Cre-positive *Kcnma1*^*fl*^*-tdTomato* siblings were intercrossed to generate homozygous *Kcnma1*^*fl*^*-tdTomato* mice (referred to as *Kcnma1l*^*fl/fl*^).

### Mice

All procedures involving mice were conducted in accordance with The University of Maryland School of Medicine animal care and use guidelines. Experimental mice were F2 Cre-positive (SM22*α*-Cre or Nes-Cre) or Cre-negative homozygous *Kcnmal*^*fl/fl*^ littermates. To harvest tissues for protein isolation and electrophysiology, mice were euthanized by inhalation of saturating isofluorane vapors, followed by rapid decapitation.

### Western blot analysis

Protein was isolated from mouse (3–4 months) urinary bladders and subjected to western blotting as described previously (White et al. 2014). BK bands were detected with 1:1000 rabbit polyclonal *α*-BK (APC-021, Alamone Labs, Jerusalem, Israel) and visualized with the SuperSignal West Dura Kit 1:500 horseradish peroxidase-conjugated goat anti-rabbit secondary antibody (Pierce). *α*-Tubulin was detected by 1:10,000 DM1A mouse monoclonal *α*-tubulin (Sigma). BK band intensity was normalized to DM1A.

### Electrophysiology recordings

For smooth muscle recordings, mesenteric artery smooth muscle cells were dissociated as described previously (Zhao et al. 2010). Potassium currents were measured with the whole-cell patch-clamp configuration with a sampling rate of 200 *μ*sec (EPC9, HEKA). The bath solution was HEPES-buffered physiological saline solution (in mmol/L: 134 NaCl, 6 KCl, 2 CaCl_2_, 1 MgCl_2_, 10 HEPES, and 10 glucose; pH 7.4 with NaOH). Pipettes (1.5–2.5 MΩ) were filled with (in mmol/L) 110 KAsp, 30 KCl, 10 NaCl, 1 MgCl_2_, 10 HEPES, and 0.05 EGTA (pH 7.2, KOH). Cells were held at −40 mV and stepped to −70 mV to +80 for 250 msec. BK currents were isolated by subtraction of the current after 5 μmol/L paxilline. For neuronal recordings, acute brain slices were prepared, and macroscopic currents were recorded in whole-cell patch clamp configuration from suprachiasmatic nucleus neurons as described previously (Montgomery et al. 2013). Neuronal BK currents were isolated by subtraction of the current after perfusion of 10 μmol/L paxilline.

### tdTomato imaging

All tissues were freshly dissected and unfixed. Bladder tissue strip images were acquired with a Zeiss LSM710 confocal microscope using a 20× objective, 561 nm excitation, and 580–703 nm emission filter. Equivalent acquisition settings were used for each genotype (Gain = 939). For analysis of fluorescence intensity, the average pixel intensities were calculated from 12 equivalently sized boxes per mouse (*n* = 3 mice). SCN images were similarly acquired from thick section coronal brain slices, prepared as described previously (Montgomery et al. 2013), using equivalent acquisition settings between genotypes (Gain = 939). For analysis, average pixel intensities were calculated from 4 to 8 equivalently sized boxes per mouse (*n* = 3 mice).

## Results

### Generation of the conditional Kcnma1^fl^-tdTomato allele

*Kcnma1* comprised 29 constitutive and 8 alternative exons, covering >700 kb of genomic sequence on chromosome 14 in mouse (NC_000080.6; NCBI Gene ID: 16531; Fig.1A) (National Library of Medicine (US), N.C.B.I. (2002). The human gene structure is conserved on chromosome 10 (NC_000010.11; Gene ID: 3778). Two prior knockout transgenic lines were generated by targeting exon 1, containing the S0 domain (Meredith et al. 2004), or exon 9, containing the pore domain (Sausbier et al. 2004).

In this study, the second exon was targeted for Cre/loxP-mediated recombination. A targeting vector was constructed using an “inversion” strategy to generate the conditional inactivation (Meredith 2015). This event consists of Cre-mediated inversion of the second exon of *Kcnma1*, with simultaneous “knockin” of a tdTomato reporter (Shaner et al. 2004) that was placed in antiparallel (reverse) orientation in intron 2. The allele retains wild-type (WT) expression of *Kcnma1* from the endogenous promotor and regulatory elements, and the reverse orientation of the 2A-tdTomato cassette will not be expressed, until Cre-mediated recombination inverts the cassette. The inversion is mediated by two mutant loxP sites, flanking exon 2 and the reverse orientation 2A-tdTomato cassette in intron 2 (Fig.1B). The mutant loxP sequences cannot undergo repeated recombination after the initial Cre-mediated inversion event. In this way, the inactivated *Kcnma1* allele is stably maintained in Cre-expressing tissues (Meredith 2015). The inversion event renders exon 2 unable to be transcribed, while bringing the 2A-tdTomato cassette into the forward orientation and placing it under the control of the endogenous *Kcnma1* promotor and regulatory elements. After transcription of the exon 1, the 2A peptide sequence mediates cotranslational cleavage via a “ribosome skipping” mechanism, allowing expression of tdTomato as a separate polypeptide (Fang et al. 2005) (Szymczak et al. 2004). tdTomato fluorescence will thus identify cells that undergo Cre-mediated *Kcnma1* inactivation.

Founder mice carrying the unrecombined allele were first mated to mice expressing FLPe recombinase under the control of the human ACTB promoter (Rodriguez et al. 2000) to remove the neomycin selection cassette (Fig.1C). Homozygous *Kcnma1*^*fl/fl*^ progeny that harbored a deletion of the neocassette were grossly normal, with no obvious health or viability differences compared to nontransgenic littermates (unpublished observations). *Kcnma1*^*fl/fl*^ mice were mated to either Nestin-Cre (Tronche et al. 1999) or SM22*α*-Cre mice (Lepore et al. 2005), and intercrossed to produce Cre-positive homozygous *Kcnma1*^*fl/fl*^ (Fig.1C).

### Smooth muscle-specific BK channel inactivation (SM22α-Cre; Kcnma1^fl/fl^)

**Figure 2:**
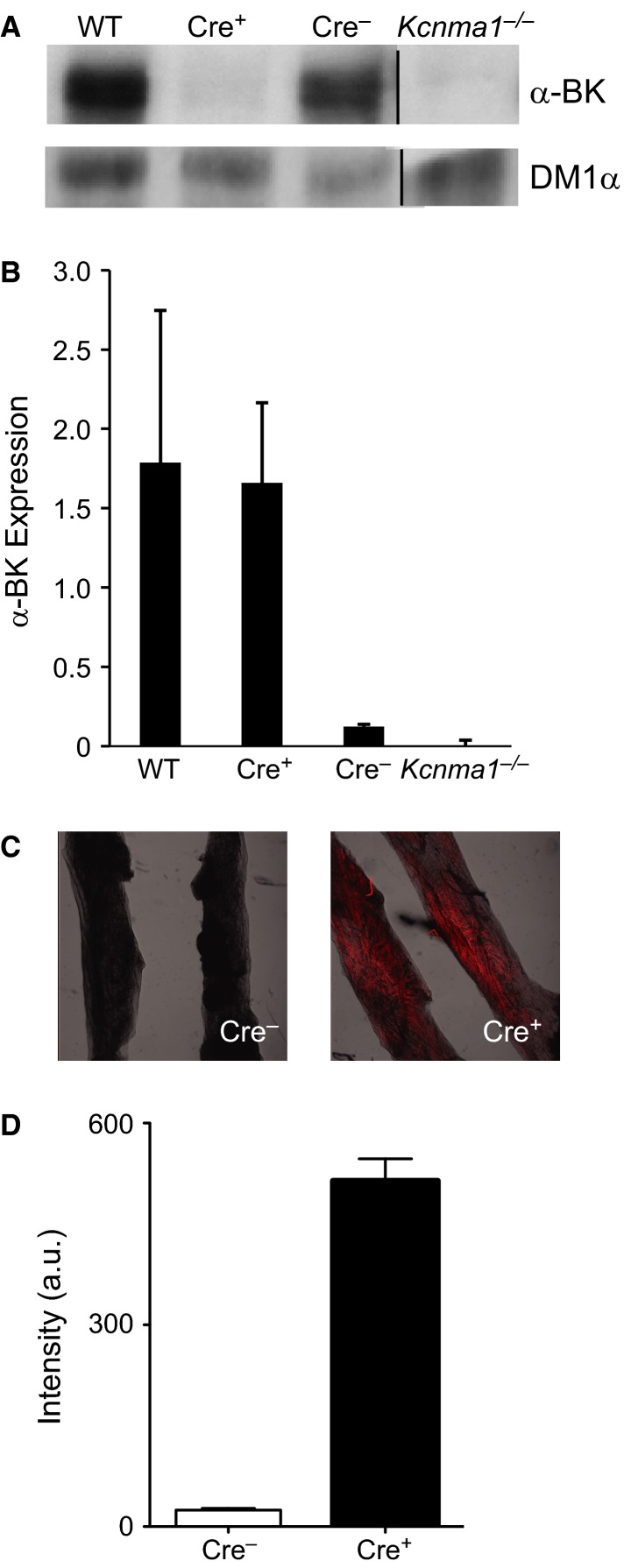
BK channel expression and tdTomato fluorescence in SM22*α*-Cre; *Kcnma1*^*fl/fl*^ tissue. (A) Representative western blot showing *α*-BK (top) and DM1*α* (bottom) expression in urinary bladder smooth muscle (UBSM) tissue from WT, Cre-positive; *Kcnma1*^*fl/fl*^ (Cre^+^), Cre-negative; *Kcnma1*^*fl/fl*^ (Cre^−^), and Kcnma1^*−/−*^. Line indicates removal of intervening lanes for ease of comparison. (B) BK expression normalized to DM1*α* (*n* = 3 bladders from independent animals in each condition). (C) Representative images from UBSM strips. Tomato fluorescence merged with bright-field image, 2.5× magnification. (D) Average tomato fluorescence from Cre^+^ and Cre^−^ UBSM strips (*n* = 3 mice for each). Data are mean ± SE.

BK channels are highly expressed in smooth muscle, especially urinary bladder (Meredith et al. 2004). Therefore, we tested the functionality of the floxed allele in smooth muscle to determine whether BK protein was eliminated by Cre-mediated recombination. BK expression was analyzed by western blot in bladder tissue harvested from WT, SM22*α*-Cre-negative; *Kcnma1*^*fl/fl*^, SM22*α*-Cre-positive; *Kcnma1*^*fl/fl*^, and *Kcnma1*^*−/−*^ mice carrying a global deletion (Meredith et al. 2004). The cytoskeletal SM22*α* promoter drives expression in most smooth muscle cells, including bladder (Lepore et al. 2005). BK expression was comparable between WT and Cre-negative; *Kcnma1*^*fl/fl*^ UBSM tissue (Fig.2A). In contrast, negligible BK channel detectability was observed in Cre-positive; *Kcnma1*^*fl/fl*^ bladder tissue, similar to levels in the previously characterized *Kcnma1*^*−/−*^ functional null mice (Fig.2A, B). This residual BK detectability in Cre-positive; *Kcnma1*^*fl/fl*^ tissue could stem from less than 100% efficiency of Cre-mediated recombination with SM22*α*-Cre in UBSM cells, or incomplete removal of the nonsmooth muscle urothelial layer, which expresses BK channels. In aggregate, these data show that BK protein expression is essentially completely gone after Cre-mediated inversion of exon 2 and confirm that in the absence of Cre expression, BK protein levels are normal.

Next to assess tdTomato expression, fluorescence was compared between SM22*α*-Cre-negative and -positive; *Kcnma1*^*fl/fl*^ bladder strips. In the absence of Cre, very little tdTomato fluorescence was observed, while Cre-positive animals exhibited strong fluorescence (Fig.2C, D). These data indicate that tdTomato is both easy to distinguish from controls and also not significantly expressed unless Cre-mediated recombination occurs.

**Figure 3:**
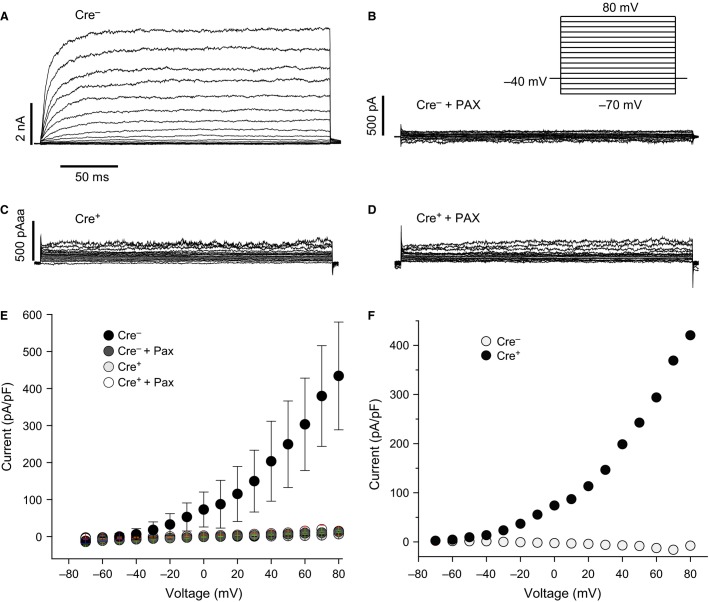
Loss of BK currents in SM22*α*-Cre; *Kcnma1*^*fl/fl*^ smooth muscle cells. (A, B) Macroscopic currents evoked in whole-cell patch-clamp recordings from SM22*α*Cre-negative; *Kcnma1*^*fl/fl*^ mesenteric artery smooth muscle cells, before (A) and after (B) 5 μmol/L paxilline. (C, D) Macroscopic currents from SM22*α*Cre-positive; *Kcnma1*^*fl/fl*^ mesenteric artery smooth muscle cells, before (C) and after (D) 5 μmol/L paxilline. (E, F) Current–voltage relationship for total outward currents (E) and the paxilline-sensitive (BK) current (F). *n* = 8 cells (Cre^−^), 3 (Cre^−^ + Pax), 7 (Cre^+^), 5 (Cre^+^ + Pax). Data are mean ± SE.

BK currents were assessed from smooth muscle cells of mesenteric artery, a cell type where the majority of the total K^+^ current is due to BK channels (Fig.3A). In Cre-negative; *Kcnma1*^*fl/fl*^ cells, large outward currents were evoked by depolarizing voltage steps, and this current was sensitive to paxilline, a BK channel antagonist (Fig.3B) (Knaus et al. 1994; Gribkoff et al. 1996). In contrast, Cre-positive; *Kcnma1*^*fl/fl*^ cells did not have large evoked currents and were of similar magnitude to Cre-negative cells after paxilline block (Fig.3C–F). These data confirm that BK currents are detectable from homozygous *Kcnma1*^*fl/fl*^ smooth muscle cells in the absence of Cre and are entirely removed when Cre is expressed.

### Neuronal-specific BK channel inactivation (Nestin-Cre; Kcnma1^fl/fl^)

**Figure 4:**
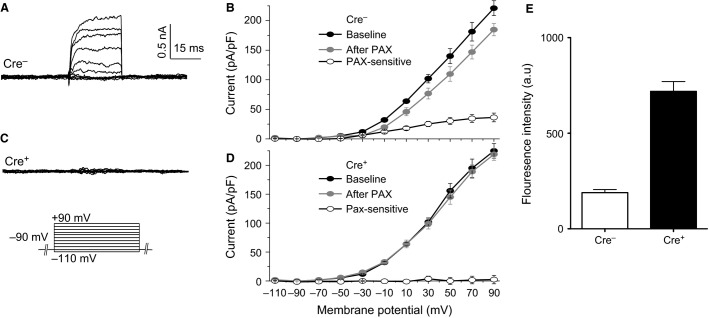
BK currents and tdTomato fluorescence in Nestin-Cre; *Kcnma1*^*fl/fl*^ brains. (A) Representative whole-cell macroscopic BK current traces. BK current was elicited with indicated voltage protocol from suprachiasmatic nucleus (SCN) neurons of Cre-negative; *Kcnma1*^*fl/fl*^ (Cre^*−*^) and isolated with 10 μmol/L paxilline. (B) Current–voltage relationships from Cre-negative; *Kcnma1*^*fl/fl*^ neurons. Total outward K^+^ current (baseline), after paxilline, and the paxilline-sensitive BK current. *n* = 6 neurons. (C) BK current from a Cre-positive; *Kcnma1*^*fl/fl*^ (Cre^+^) neuron. (D) Current–voltage relationships from Cre-positive; *Kcnma1*^*fl/fl*^ neurons. *n* = 6 neurons. (E) Average tdTomato fluorescence from Cre^−^ and Cre^+^ SCNs (*n* = 3 slices from independent animals for each). Data are mean ± SE.

We further confirmed the ability of Cre recombinase to recombine the *Kcnma1*^*fl*^ allele in neurons using a Nestin-Cre mouse line that expresses Cre throughout central and peripheral neurons (Tronche et al. 1999). We recorded macroscopic BK currents in whole-cell patch-clamp mode from neurons of the suprachiasmatic nucleus (Montgomery et al. 2013). In neurons, the BK current comprises a smaller component of the total K^+^ current. Paxilline-sensitive BK currents were present in Cre-negative; *Kcnma1*^*fl/fl*^ SCN neurons (Fig.4A, B), but not in Cre-positive neurons (Fig.4C, D). tdTomato fluorescence was higher in Cre-positive neurons than Cre-negative controls (Fig.4E), although some red fluorescence was detected in Cre-negative; *Kcnma1*^*fl/fl*^ slices and likely stems from autofluorescence (Schnell et al. 1999). These data corroborate the functionality of the *Kcnma1*^*fl*^ allele in a second tissue.

## Discussion

The goal of this study was to demonstrate the null functionality of the *Kcnma1*^*fl*^ allele and the expression of tdTomato in the presence of Cre recombinase expression. Neither smooth muscle- (SM22*α*-Cre) nor neuron-specific (Nestin-Cre) BK channel deletions exhibit gross developmental, viability, or neurological (ataxia or tremor) phenotypes (unpublished observations). This is in contrast to the global BK channel deletion mice (*Kcnma1*^*−/−*^), which exhibit increased mortality, ataxia, and tremor (Meredith et al. 2004). The loss of BK currents and protein, and corresponding expression of tdTomato, was demonstrated in Cre-positive mice. BK protein and currents were grossly comparable to WT levels in Cre-negative mice. These results indicate the utility of the *Kcnma1*^*fl*^ mouse line in further studies requiring tissue-specific deletion of the BK channel.
